# Benefits and functionality of an interorganisational workplace health management network – insights from the companies’ perspective

**DOI:** 10.3389/fpubh.2024.1380032

**Published:** 2024-07-24

**Authors:** Luisa Hente, Torsten Schlesinger

**Affiliations:** Department of Social Science of Physical Activity and Health, Institute of Human Movement Science and Health, Chemnitz University of Technology, Chemnitz, Germany

**Keywords:** workplace health management, interorganisational networks, functionality, small and medium-sized enterprises, case study

## Abstract

**Introduction:**

Workplace health management (WHM) is a worthwhile investment for companies. Nevertheless, the implementation of health-promoting interventions remains limited, especially in small and medium-sized enterprises. Interorganisational networks could be a promising way to raise awareness of the advantages of implementing WHM. Therefore, the aim of this study is to analyse the perceived functionality and benefits of a regional WHM network from companies’ perspective and to present initial results on this specific topic.

**Methods:**

An explorative qualitative case study was conducted analysing ERZgesund, a WHM network in a rural region in Germany. Twenty-two companies that participated in the network were interviewed about their experiences and perceived advantages and disadvantages participating in the WHM network ERZgesund.

**Results:**

The findings show that the network has raised awareness about WHM among the companies, provides opportunities for exchange of knowledge and experiences, and generates or strengthens collaboration. The positive effects were enhanced by the network’s structure, such as regionality and a direct contact person. Nevertheless, some companies stated that they would welcome a higher level of participation and transparency.

**Conclusion:**

Overall, it becomes clear that a WHM network can be a valuable tool to emphasize the relevance of WHM to companies. Therefore, further studies should validate and intensify the research on WHM networks to ensure a long-term benefit from the network.

## 1 Introduction

The purpose of the Prevention Act of 2015 is to strengthen the effectiveness of health promotion and prevention in Germany (1). In this context, the workplace and therefore a company is an important central setting for prevention interventions due to the fact that a large number of people [75.6% employment rate; 45.6 million employees (2)] can be covered by workplace health management (WHM). On the one hand, health-promoting interventions can strengthen and improve employees’ health status and address health risks (3–6). On the other hand, workplace health promotion (WHP) is associated with other valuable benefits such as higher job satisfaction and well-being, increased motivation, improved working environment and stronger identification with the company (5–7). In addition, WHP enhances the company’s image as a considerate employer (8–10). These positive effects generally result in lower absenteeism and sickness-related costs and safeguard or increase the company’s productivity and profitability (10–12). This suggests that WHP is a worthwhile investment for companies, particularly against the background of a number of challenges such as increasing mental stress, an ageing workforce and the shortage of skilled workers (13–17).

Despite these challenges and the positive effects of health-promoting interventions, the number of companies—and hence of employees—covered by WHM remains low (16). Only 27% of companies in Germany stated that they implement or financially support interventions to promote employees’ health (17). Furthermore, there is a discrepancy between company size and the level of WHP implementation. Small and medium-sized enterprises (SMEs), which make up about 99% of all companies in Germany (18) and contribute considerably to economic growth (19), face significant challenges in implementing WHP interventions and are underrepresented in this field. A lack of organisational capacities including financial, time and personnel resources as well as infrastructural requirements are some of the major hindering factors (12, 20–23). Other obstacles to the implementation of WHP include insufficient know-how and competences in the field of WHP, as well as a negative attitude towards (workplace) health (9, 12, 16, 20, 21, 24).

The establishment of interorganisational networks seems to be a promising and suitable option for addressing these obstacles, especially for SMEs (16, 20, 25). Networking offers tremendous potential, such as the bundling of competences and the exchange of experiences and information. In addition, resources (financial, time and personnel) can be utilised efficiently and synergies created, which increases overall effectiveness and benefits all participants involved (leading to win-win constellations) (16, 20, 26–28).

Despite widespread recognition that interorganisational networks are a promising avenue to WHP in SMEs, not much empirical data are available on the advantages of WHM networks for companies. According to Schäfer et al. (29), over 50% of companies expressed interest in participating in a WHP network. The most widely stated reason is mutual exchange. According to Müller et al. (30), 88.1% of network members gain benefits from the exchange of experiences and 50% from the advantages of networking. Hente et al. (31) analysed the processes and structures of a WHM network based on a case study and identified several facilitating and hindering factors that influence the establishment and development of a WHM network. However, no studies are available that explicitly address the functionality and effectiveness of a WHM network from companies’ perspective. Focusing on the companies’ expectations and needs within WHM network are important, because as a participating actor they play a key role in shaping and promoting the development of WHM networks. Conversely, unfulfilled expectations or unfavourable cost–benefit ratios are associated with less commitment or even abandonment of the network, which in turn weakens the network as a whole. The expectations and objectives of companies involved in a WHM network should therefore be taken into consideration.

This article examines the functionality and benefits of a regional WHM network from companies’ perspective based on a case study. The following research questions are explored in more detail: *What are the advantages and disadvantages (perceived functionality) of participating in a regional WHM network from the companies’ perspective?*

## 2 Methods

### 2.1 Contextual background

This analysis focusses on the WHM network ‘ERZgesund – Gesunde Unternehmen im Erzgebirgskreis’ [eng: ‘ERZgesund – Healthy Companies in the Ore Mountains’]. It was developed in the Ore Mountains, a district in the southern part of the Federal State of Saxony (Germany). With 344,136 inhabitants (115,753 employed persons), the district has a population density of 183 inhabitants/km^2^ (32); the average in Germany is 233 inhabitants/km^2^ (33). Demographic change is noticeable in this region due to the migration of younger people to western parts of the country and/or to larger cities. With an average age of 49.1 years, the region’s population is among the oldest in Germany (34), and the region’s ageing workforce and lack of young skilled workers pose serious problems for companies. The majority of 15,363 enterprises (87%) in this region are micro-enterprises (1–9 employees). Small enterprises (10–49 employees) account for 10.7%, medium-sized enterprises (50–249 employees) for 2.4% and large enterprises (≥250 employees) for 0.2% of total enterprises in the Ore Mountains.

Due to the region’s rural structure, its traditional orientation and company size structure, over 60% of companies have not implemented any WHP measures (35). Discrepancies related to company size have been also identified. For these reasons, the WHM network ERZgesund was established by a trade association (Industrie- und Gewerbevereinigung Aue; IGA) in 2017 with the aim of raising awareness of WHM, particularly among smaller companies, to open opportunities to implement WHM, share experiences and knowledge, create a service catalogue and to design and award companies with a health seal. In addition to both health promotion and maintenance, the network further sought to address demographic change, secure skilled workers and to strengthen the region in general.

**Figure 1:**
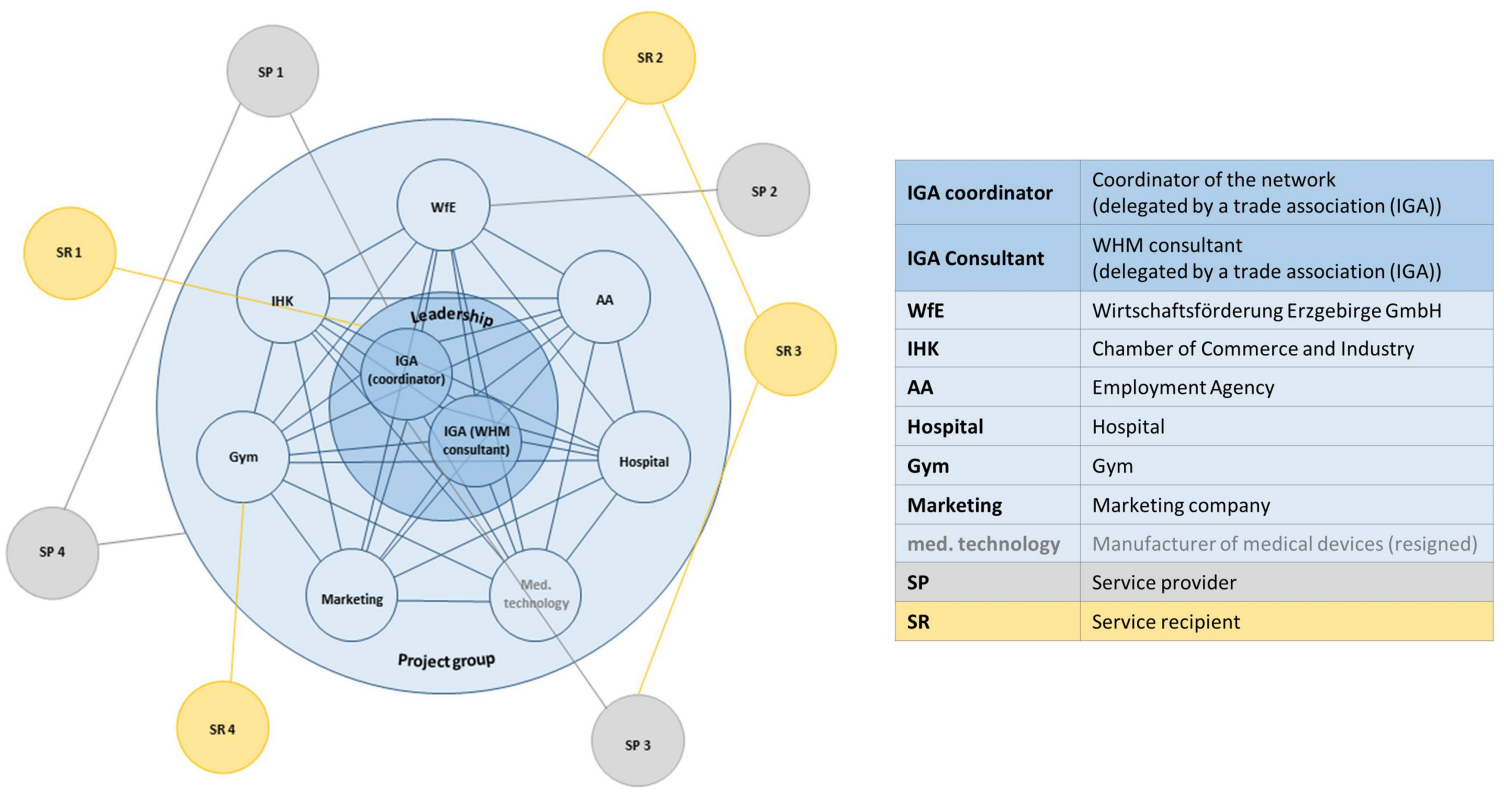
Exemplary visualisation of the ERZgesund network structure: the project group consisting of the leadership (inner blue circle) and the project partners (light blue circle); SP (service provider; grey), which either has connections with (individual) members of the project group, the entire project group or with SR (service recipient) or other SP; SR (yellow), which has connections with individual members, the entire project group or with SP or other SR.

The leadership, namely the IGA coordinator and a WHM consultant, act as the nucleus of the network. Together with six other authorities, companies and individuals from the public and private sectors, they represent the project group and are in charge of the network’s structural design and development. In addition to the project group, the network consists of the service recipients (SR) and service providers (SP). During the funding period (2017–2019), a total of 19 events were organised where over 300 different companies (SP, SR) participated. The service catalogue, which lists the SP, shows that six companies offer general WHM services while 51 companies provide individual health-related services, such as stress management, exercise/physical activity, addiction prevention, nutrition, education and training, etc. Despite the relatively strong relationships, the network has a flexible hybrid structure, reflecting the voluntary and individualistic participation of the companies. Figure 1 presents the structure of the ERZgesund network.

### 2.2 Sampling and data collection

A qualitative case study design was used to analyse the perceived functionality of companies as participants in the WHM network ERZgesund (36). A case study design makes it possible to capture in-depth insights about organisational experiences in their real-world contexts [e.g., (37)]. Thus, the case study design approach conducted in this study enables the investigation of exploratory questions concerning the “what” and “how” (37) regarding the experiences of companies participating within a WHM network. This approach makes it possible to identify aspects (“what”) companies consider as relevant in WHM network participation as well as to analyse how companies perceive these aspects – as beneficial/functional or dysfunctional from their point of view and against the background of their own goals and expectations (“how”).

Problem-centred interviews were conducted with 22 companies participating in the network. Statistical representativeness was not the aim of this qualitative case selection, instead, it was sought to include as many different cases as possible in the survey to represent different structural characteristics (38). The companies were selected according to the principle of contrasting [e.g., (39)] in terms of sector, size, role/function in network and their participation frequency (see Table 1). Participation in the interviews, which took place between October 2020 and March 2021 with 23 authorised persons from 22 companies (in one interview two persons were present), was voluntary (informed consent was obtained) and lasted between 25 and 80 min. The interviewed persons were company executives in positions related to WHM and who usually also represent the company in the ERZgesund network.

**Table 1:** Overview of the companies surveyed by industrial sector, size and position in the respondent’s company as well as the company’s function in the network [service recipient (SR) and/or service provider (SP)] and participation frequency (one time, rarely, frequently, regularly).

Code	Industrial sector	Size	Position	Function	Participation frequency
U01	Manufacturing industry	Medium enterprise	Chairman of the company	SR	Frequently
U02	Service	Micro-enterprise	Owner	SP & SR	Regularly
U03	Sales	Medium enterprise	Assistant Manager	SP & SR	Regularly
U04	Construction	Small enterprise	Director	SP & SR	Regularly
U05	Service	Micro-enterprise	Owner	SP & SR	Regularly
U06	Construction	Small enterprise	Sales representative	SR	One time
U07	Service	Micro-enterprise	Owner	SP & SR	Frequently
U08	Service	Micro-enterprise	Owner	SP & SR	Frequently
U09	Manufacturing industry	Medium enterprise	Personnel Officer	SR	Rarely
U10	Manufacturing industry	Large enterprise	Assistant personnel manager	SR	Rarely
U11	Health and social services	Micro-enterprise	Owner	SP	Frequently
U12	Public administration	No information	Team leader	SR	Rarely
U13	Manufacturing industry	Small enterprise	Managing Partner	SR	Frequently
U14	Water supply	Small enterprise	Public Relations	SR	Rarely
U15	Service	Micro-enterprise	Director	SR	Rarely
U16	Service	Micro-enterprise	Co-partner	SR	Rarely
U17	Service	Small enterprise	Director	SR	Rarely
U18	Manufacturing industry	Medium enterprise	Personnel Manager	SR	Frequently
U19	Sales	Micro-enterprise	Owner	SP & SR	Rarely
U20	Manufacturing industry	Large enterprise	Company Nurse & Personnel Manager	SR	Regularly
U21	Motor vehicle	Medium enterprise	Service Manager	SR	Rarely
U22	Manufacturing industry	Small enterprise	Personnel Manager	SR	Regularly

The interviews followed theory-driven and semi-structured guidelines covering specific topics, while ensuring sufficient openness (40). The interviews addressed the status of WHP in the company, the structure of and participation in the network, and the network’s effectiveness, benefits and problems according to the company. In addition, companies’ structural characteristics were investigated. Table 1 presents the companies with some characteristics, and the position of the interviewed person within the company.

All 22 interviews were recorded and transcribed with the participants’ consent. By taking part in the interview, the interviewees also agreed that their statements would be further processed in anonymized form for analysis purposes, which also includes publications. The transcripts were anonymised and coded, and thus, no conclusions can be drawn about the individual participants. The responses were evaluated using a qualitative content analysis according to Mayring (41), with the aim of processing the material in a structured way by coding all relevant interview passages in a system of categories. The main categories and subcategories were determined in advance based on theory (deductively). The categories were used to describe networks and their success and included regionality, coordination, participation, exchange of experience, relationships, effects, etc. (25, 42–46). Text passages that could not be assigned to a deductive category were grouped into newly created inductive main categories or subcategories. The coding scheme was therefore supplemented by additional categories that emerged from the responses. The text material was coded using MAXQDA software. Quotes from the interviewees were translated into English to better exemplify the results of the analysis.

In addition, a quantitative analysis was conducted, where the codes were labelled with frequencies to illustrate the quantity of the codes in the 22 interviews. The frequencies include not only the exact wording but quiet similar content of statements. The presence and frequency with which certain benefits of the ERZgesund network are mentioned is seen as an indicator of the relevance attributed to a specific benefit in the context of the respective company (47).

## 3 Results

First, the expectations of the network are presented from the participants’ perspective in order to be able assess the estimations towards the network better. In a second step, specific categories are used to discuss the network’s advantages and disadvantages from companies’ perspective. Subsequently, suggestions for improvement and the preferences of the network participants are outlined. The data obtained are summarised in Table 2.

**Table 2:** Advantages, disadvantages and opportunities for improvement of the ERZgesund network from the participants’ perspective [in brackets: the number of mentions in the interviews].

Category	Advantages	Disadvantages	Improvements
Regionality and physical proximity	Size of the region in general [16] Short distances [7] Feeling of belonging and familiarity [10] Added value [19] Developing and strengthening the region [19]	West Ore Mountain focussed [6] Not adequately represented region/ regional imbalance	Possible expansion [6] Non-IGA companies (Trade) associations
Contact person and right to have a say/participate	Direct contact person [19] Committed and passionate project group [17] Personal interactions [15] Taking time for each and every one [3] Coordination in the background [19] Free expression of opinions [7] Opportunity to participate and design [14]	Top-down approach [5] Project group determines topics (not aware that getting involved was a possibility)	Requests for topics and expert presentations [5]
Interaction	Casual [17] Open [17] Pleasant [17] Contact without apprehension [13] Harmonious [16] Familiar [10] Good atmosphere [15]	Minor tensions within the project group [5] Personal differences	
Investment of time	Absolutely appropriate (not too long, not too short) [18] Not mandatory [14] free choice in suitable events (Event) distribution [5]	Too many events [3] Cause for stress [2]	
Diversity	Different companies [19] Events [19] Varied Individual Innovative Learn new things [17] Locations [14]	Similar events [3] Lower interest Lower participation Lack of specialisation (topics) [5]	
Exchange of information and experiences	Speakers [8] Professional exchange Content [20] Solid and modern design [9] No own investigation/research [10] Exchange of experiences [19] Practical examples [18] Homepage [3] Catalogue of services [8] SR (health providers/services at a glance) SP (publicity/ advertising)	Not all information was relevant [4] Little content and exchange opportunities for companies that have already implemented WHM more comprehensively [5] Homepage was not always up to date [2]	Experience exchanges with other WHM networks [6] Include more WHM experienced companies [6] New and special topics [10]
Influence on WHM measures	Raising awareness/ sensitisation [20] New impulses [19] Structure based on health pillars (contents) [3] Measures implemented [11] Comparison with other companies [4]	Health providers/services were not used [8] COVID-19 pandemic limited implementation [11] Financial costs for WHM [6]	
Contacts and relations	New contacts established [22] Existing contacts/cooperation structures could be intensified [11] Partial cooperation [7] Reaction chain [6]	No or barely any cooperation [10]	
(Termination of) project funding of the ERZgesund network	Intensive phase (funding period) [11] No changes required [6] Optimistic expectation [10]	No information about network continuation after the project duration and during the COVID-19 pandemic [17]	Information on the future plan after the end of the funding period and during the COVID-19 pandemic [18] Marketing and sales [10] Overview of events [15] Possible contribution was considered [4]

### 3.1 Expectations of ERZgesund network participants

The majority of participating companies had no explicit expectations of the ERZgesund network.

“And of course I approached it with positive expectations because I was just curious. Because I had never taken part in something like this before, because WHM had not really existed in our company before” (U12, 22).

“No, I actually had no expectations at all. I generally let myself be guided a bit. That is, like, what lies ahead for me? It’s difficult to have expectations if you have not done anything like this before” (U14, 32).

Most respondents hoped to establish new contacts through the ERZgesund network and thereby expand their own network, as well as to learn more about WHM and benefit from exchanges of experiences.

“Mainly to establish contacts to expand our network” (U06, 28).

“First of all, building up contacts. […] My goal was actually […], via the network, to find out what’s new on the market” (U05, 15).

“Establishing contacts, being inspired” (U21, 69–70).

“Just learning, just gaining experience [...] an awareness” (U03, 43).

Individual companies saw their task within the network as raising awareness and sharing experiences.

“We considered ourselves more as an experience provider for companies that are just getting started” (U20, 68).

### 3.2 Advantages and disadvantages of the ERZgesund network

#### 3.2.1 Regionality and physical proximity

As a regional network, ERZgesund is characterised by a spatial agglomeration of the organisations that belong to the network. Accordingly, the aspect of regionality was highlighted as a particularly important factor, albeit for different reasons. Regionality in terms of the region’s size and thus the short distances, as well as the companies included in the network were mentioned.

“Well, above all the regionality. I would say that is a huge plus point. It’s not just some organisation from Hamburg or something. But that’s what happens here. That regional partners are also involved” (U17, 58).

“No, it was really ideal in this framework [Ore Mountains]. Everything else would have simply been too far away, I think. Not only regionally, but it would have just gotten too big” (U22, 58).

The feeling of belonging and familiarity and the added value generated by the ERZgesund network for the entire region as a whole were frequently stated. The network contributes to the development and promotion of regional identity and encourages as many companies as possible to closely collaborate because they are all equally “affected.” The connection between the companies with the region and the network’s goals serve as an additional incentive to participate in it. Moreover, it was noted by the respondents that the network unites and strengthens the region.

“The tiny bit of “us-feeling” is already positive [...] Um, of course it also brings an, I’ll say an improvement to the sense of belonging in the region” (U13, 62).

“It has a very good added value for the region” (U05, 77).

Nevertheless, for some companies it was “slightly west Ore Mountains-pronounced” (U08, 76). That is, the entire region was not adequately represented and there was a regional imbalance.

Some companies also noted that the network could have also been extended beyond the Ore Mountains. This would have offered the opportunity to think outside the box and learn from the experiences of other networks or companies.

“And why should not we now look at Saxony, for example, and see what progress has already been made there in this area? So, I would have welcomed that” (U12, 66).

#### 3.2.2 Contact person and the right to have a say/participate

The fact that the network has a direct contact person and that the project group as the coordinating authority was involved with “commitment” (U08, 76), “emotion” (U20, 32) and “persuasion” (U12, 62) were positively highlighted. Network coordination is based on trust, partnership and socio-cultural proximity. The coordinator generally maintained very close personal relationships with participants and invested time in them. In addition, many coordination activities were carried out behind the scenes without having to disturb the participants.

“I have to say, what I really liked were the coordinators, who were extremely personable. I spoke to both of them personally and always had the feeling that they took the time to listen. And of course, they did a lot of coordination work behind the scenes, which was not very perceptible to the outside world” (U11, 50).

The respondents’ views on their right to a say and participation diverged. On the one hand, some companies criticised the project group’s top-down approach and degree of control. The companies claimed that the project group chose the topics without involving the participating companies. On the other hand, others stated that companies in the ERZgesund network were given the opportunity to actively participate and contribute. They asserted that “everyone could give their input” (U05, 26) or that more active involvement would have been possible. Some companies also helped organise the events and were part of them.

#### 3.2.3 Interaction within the network

The success of networks and their integrative logic of action depends on no one being in either a dominant or a completely powerless position. The management and balancing of interactions, relationships and social influence potential within the network are therefore crucial. The relationships and interactions within the network were “relaxed,” “very open, very pleasant” and without “reluctance to contact others” and was perceived as “harmonious” (U16, 40; U19, 28; U01, 78). It was “already familiar, because you knew some people from the region. Half of them on a “first-name basis, half on a second-name basis” (U03, 91). There was also a “positive attitude” towards the project group (U20, 74).

Only minor tensions were felt within the project group. “So I did notice that there was a bit of a hitch now and then or something. But I think that’s just the way it is. Whether you are in a club or in a company, there’s always someone who wants to go in a different direction. But it’s not like it was extremely disruptive. Well, at least that’s not how I felt about it” (U13, 60). Nevertheless, the majority of companies described the coordination and any changes as “fluid” (U03, 133).

#### 3.2.4 Investment of time

Concerning the investment of time during the funded project period, most companies stated that “it [was] absolutely appropriate. It was not too much, it was not too little” (U09, 30). Nevertheless, some participants also pointed out that if too many network events had taken place, it could have become quite stressful. Moreover, some companies asserted that they did not participate in all events or that they divided participation in events among company staff. The network events were not mandatory. The participants were able to choose the events that suited them in terms of time and content.

“Well, by not attending all of them, in the end, I set my own timelines. Well, I’d rather go to a few too many than to too few” (U14, 60).

“It was a healthy balance. It did not become a burden for anyone. And as I said, we managed to divide up participation” (U13, 40).

#### 3.2.5 Diversity

The ERZgesund network plays an important mobilisation function to overcome the limitation to sectoral perspectives in favour of common health-related concerns. In this context, the network’s diversity was also commended. On the one hand, the respondents referred to the different companies, “the diversity of companies across the board, that was definitely an advantage for me [...] this cross-section is definitely good. It’s healthy for such a network. Because diversity plays a role, you never actually concentrate on just one, because everyone is facing the same problem” (U01, 68).

In addition, the network’s diversity facilitated the search for new solutions and courses of action. Companies mentioned that the network events had an important forum and innovation function, which were described as “varied and individual” (U20, 88) and “very innovative” (U12, 62). As a result, the companies “always learned something new and became familiar with a few locations” (U02, 22).

Despite the stated benefits of the diversity of network events, a lack of more specialised topics was reported: “that we specialise in [...] for example a shift system” (U01, 74). One company also commented that “the second event was similar to the first one. That does not make sense. I mean, for me it was a waste of time” (U17, 38). This reduced the interest and participation of some participants in the events.

#### 3.2.6 Exchange of information and experiences

Topic-related networks offer valuable opportunity structures for the exchange of experiences and thus foster learning processes. The majority of surveyed companies were very satisfied with the level of professional exchange. The speakers, content and design of the events were appreciated.

“Technically, [it was] also very good. [...] You always had highly reputable speakers, which was fitting” (U03, 93).

“The professional exchange was terrific [...] really terrific, because on the one hand, I had the feeling that the right people had been invited to the right place” (U11, 54/56).

“We do not want to invest too much effort into it, we want to more or less have it served to us and it should be a bit fresh and modern and that is one of the great strengths of this network” (U11, 78).

Furthermore, enough information was provided “without being flooded with information right from the start” (U02, 103). Nevertheless, not all the information was necessarily relevant for the companies: “But you do not always learn something new after such evenings and whatever information I did not pick up, anything that did not interest me, I simply left it there. But I actually took away a lot more than I had expected” (U11, 102).

The companies also exchanged their own experiences, however, this depended on the extent to which the companies actively promoted the health of their employees.

“I found it very useful that a lot of work with practical examples was presented or by individuals who had the corresponding experience” (U12, 40).

“So the advantage is definitely the exchange with other companies” (U18, 56).

“It is through the network [that you realise that] it is crucial to motivate and have a bit of a vision and then you also realise that it is important to keep your company fit in terms of health” (U19, 10).

This exchange of experiences played an important role for many companies to obtain important impulses and ideas for health promotion and at the same time to neutralise uncertainties. Nevertheless, some of the companies that have already implemented health-promoting interventions reported that there had been insufficient exchange and information on specific topics and questions: “That we get more inputs on what possibilities we have to expand our measures” (U20, 22).

The homepage including the catalogue of services was perceived as an important exchange platform for both SP as well as SR. “Information was made available” (U19, 20) and “SR [could see] very many SP and thus also find a potential solution for their issue” (U07, 68). SP, on the other hand, gained visibility and access to a larger number of clients at the events (*cf.* U07, 70). However, the information did not always seem to be directly accessible on the homepage: “That everything just apparently took a bit longer than desired” (U14, 82).

#### 3.2.7 Influence of the ERZgesund network on WHM measures and contacts

In addition to the perceived (dis)advantages, the question arises to what extent the companies were able to roll out or implement specific WHM interventions and projects in their company as a result of their ERZgesund participation and whether they were able to establish new contacts and collaborative activities.

Many companies were sensitised by the network: “And, of course, sensitisation for the topic” (U16, 62). New impulses were set and health promotion in the different companies could be structured around the pillars (*cf.* U11, 8; U18, 24). The implementation of measures within companies as a result of network participation varies considerably. Some did not make use of any of the offers provided, nor did they implement any measures (*cf.* U02, 91). In those cases, the interest, relevance and resources were insufficient to introduce or implement further health-promoting interventions. Others, on the other hand, wanted to introduce interventions, but were not yet able to do so due to the COVID-19 pandemic or for financial reasons.

“Yes, unfortunately, due to the corona situation, as I said, from a more practical point of view, our hands are very much tied. We had a lot of plans, but we will definitely implement them in the future. [...] So it was definitely a help and good support” (U09, 36, 38).

“Theoretically, yes, practically, um, I’ll say less so. [...] But I say this because it is associated with certain costs. So generally speaking” (U05, 48).

On the whole, only few companies were able to roll out or implement health-promoting intervention in their company through the network and its impulses. However, the companies acquired missing information and were able to fill knowledge gaps, and received advice from the network and its participants. They were furthermore introduced and could commission SP. The companies were interested in promoting the health of their employees and were prepared to spend (financial) resources on their employees’ health. One of the companies that participated in the survey achieved a lot with the help of the network (*cf.* U22, 8, 12, 34, 42, 44). The network sensitised the company and informed about the benefits and advantages of WHM. The network’s structures and topics were used to improve the working environment and to introduce health-promoting interventions. Finally, the company integrated health into its culture.

Another company realised by being part of the network that it was actually already well positioned: “We often went to the events and realised that we are already on the right path! So, many things were addressed there. And we could say, sure we’ll do that!” (U20, 58). Nevertheless, it is important to note that the network can only lay the foundation by imparting knowledge and supporting implementation. The decision to implement health-promoting interventions lies with the companies themselves (*cf.* U03, 109; U20, 70).

The statements made on contacts and cooperation were also contradictory. All companies confirmed that new contacts had been established. They did not, however, know whether true cooperation with these contacts would ensue (*cf.* U2, 99). Other companies, by contrast, had already “been able to establish new cooperation structures through the network” (U15, 37–38). Furthermore, relationships with existing contacts or cooperation structures intensified through the network (*cf.* U14, 72). One company described networking as a reaction chain: “If I had not known about the whole ‘Ore-Healthy’ project in the first place, I would not have got the order [...] there and there, and so on. It’s a reaction chain, you cannot even know what you’ll get out of it in five years” (U03, 123).

#### 3.2.8 Suggestions for improvement

Finally, suggestions for improvement of the network and recommendations were expressed.

Most companies had not yet heard back from the network at the time of the survey due to the COVID-19 pandemic and the end of the funded project period. Many respondents stated that they would have welcomed if the network had at least contacted them to inform them about the status and provide some updates (*cf.* U15, 74). Some companies had no suggestions for improvement, they “would do it all over again the same way” (U03, 153). Others stated that they would like to expand the network. First, “not only the companies, but also perhaps other associations” could be included in the network (U17, 70). Secondly, companies “outside the IGA” should also be more involved (U08, 30). In addition, marketing as well as sales and distribution channels ought to be improved (*cf.* U05, 77; U07, 90; U14, 90). Expert presentations on specific topics (*cf.* U13, 58) as well as innovations and special topics should also be included (*cf.* U20, 72). In addition, it should be possible to view an overview of the following dates (*cf.* U18, 58).

The companies generally had optimistic expectations about the continuation of the ERZgesund network (*cf.* U03, 165).

“Yes, once Corona is over, it would be nice to have another network event, really nice. Such events will certainly no longer happen in the same frequency as in the initial intensive phase during the 3 years it ran as a funding programme. But I’m sure something will come up. And no one will shy away from making a contribution of 20 euros to support such a networking event” (U02, 150).

Table 2 summarises the results of the survey.

## 4 Discussion

This qualitative case study analysed and discussed companies’ perspectives of a WHM network. The study’s overall aim was to determine how beneficial and functional the WHM network ERZgesund is for the participating companies. The results show that the majority of companies perceive the ERZgesund network as a *useful tool for raising awareness and implementing health-promoting interventions* in companies that aim to address the region’s most serious challenge – demographic change and the associated shortage of skilled workers. The underlying reason is the shared goal of keeping employees healthy and to thus increase the companies’ profitability and to strengthen the region, since a common goal or purpose of a network is decisive for its success (45, 46, 48). In addition to a common goal, trust within the network and commitment are indispensable (46, 49, 50). A certain level of trust within the network existed from the beginning due to the pre-existing *contacts and relationships* between the participating companies. This may also be attributable to the *size of the region* and the companies’ affiliation and familiarity with it. Nevertheless, some companies criticised that the network did not extend to the entire Ore Mountain region, and instead focussed on the region’s western area. This was mostly due to the location of the coordinating authority. It would be conceivable to extend the network to the entire region or to subdivide the region so meetings can take place in a similar and timely manner and meetings for the entire region only occur occasionally. Accordingly, the proximity of meetings would reduce the amount of *time commitment* and the level of awareness, participation in the network and the exchange of experiences within it could increase. It would be difficult, however, to expand the network beyond the Ore Mountain region, as this would imply that it no longer is a regional network *per se* with a common purpose, facing similar problems and characteristics. Moreover, the companies described the network and the *relationships and interactions* between the members as very open and easily accessible and commended the level of familiarity and good atmosphere, which enhanced the degree of trust between each other. If the network area were expanded, the level of proximity and familiarity as well as the general atmosphere could suffer. It would be worthwhile for the network to connect with other WHM networks nationally or perhaps even internationally, to promote further exchange of experiences and to increase diversity.

On the one hand, engagement and commitment was demonstrated by the participating companies, which contributed and proposed topics, helped develop ideas or made their company available as a best-practice example and location. On the other hand, the commitment and presence of the project group as the coordinating authority were appreciated. A direct *contact person* was available to answer questions and made important decisions regarding the network. Even though the network’s structure is relatively hierarchical with the coordinator at the top, the project group and participating companies, contrary to existing literature (49), the structure seemed to have been beneficial for the purpose of the network. This corresponds with newer studies that also points out that a WHP network should be actively managed (51). To increase awareness of health promotion, it is important to have expertise in both coordination as well as in health-related content to effectively promote exchange and knowledge transfer within the network (44, 45). The *exchange of experiences*, which is a key component of networking in addition to the creation of synergies (25, 42, 43), was described by the companies as very useful and positive. The companies did not have to research information on the issue themselves, but absorbed the information they considered useful and thereby saved time. The companies did not have to contribute financially during the project phase, they only had to invest the time they spent participating in events. Only companies that already have a more advanced WHM would have preferred more specific opportunities for exchange and expertise. The network should include more experienced companies in the field of WHP and involve health professionals.

The high number of events during the *funding phase* meant that many companies were reached, with repeated opportunities to raise awareness of WHP. Even if some companies felt that too many events had been organised or that it was too stressful, participation in the events was voluntary.

The topics and *diversity* of the events were appreciated, although some companies stated that the events were too similar. One reason for this could be that the network consists of relatively stable relationships, which is a positive thing for a network (52), but wanted to expand the network further to intensify the exchange of experiences and to sensitise more companies. Thus, some basic information was the same at all of the events. Nevertheless, to further develop the ERZgesund network, the expectations of companies should be considered in more detail, as participation and equal interests are indispensable for maintaining a solid long-term relationship (53).

The network’s impact on individual companies has differed considerably. In terms of *relationships*, all companies were able to establish new contacts and exchange information, even though not all of them had established new cooperation structures at the time of the survey. The companies were sensitised to *WHP* and their implementation and received valuable impulses for initiating implementation. Some companies were able to establish or strengthen WHM, while others were not yet able to roll-out interventions due to financial restrictions, the COVID-19 pandemic or other reasons. (Financial) resource allocation seems to be a major obstacle for the implementation of health-promoting interventions despite the existence of a network (12, 20, 21). Nevertheless, the first step of raising awareness among companies and the associated knowledge transfer, which is also crucial for implementation (20), was ensured by the network, since many companies had no or little experience with WHP prior to joining the network.

According to Raab (54), interorganisational networks are important on three levels. Firstly, the network influences the position of the organisations within the network in terms of new knowledge and general opportunities and constraints. This is also the case with the ERZgesund network. It provides knowledge and highlights the possibilities and limitations of WHM. Secondly, how the network is structured and managed has considerable influence on the results and its functionality. As already mentioned, the ERZgesund network was relatively hierarchical, and the topics were mostly predetermined by the project group. Not all participating companies were aware that they could actively get involved and contribute. The exchange of information after the end of the project phase and during the COVID-19 pandemic was especially criticised. The companies would have welcomed more transparency in this regard. Overall, it can be concluded that the companies would have preferred to be more closely involved. The pandemic, in particular, was a major challenge for the companies, which not only affected health promotion, but also their general business processes and thus their economic efficiency (55, 56). Third, interorganisational networks are beneficial for society. The ERZgesund network seeks to positively influence society by promoting health promotion and strengthening the region by addressing the shortage of skilled workers and improving its image.

The network is generally a valuable tool to raise awareness of WHM and to benefit from the exchange of experiences and knowledge as well as the relationships formed. Nevertheless the decision to implement WHM still remains with the companies themselves, as a manager stated: “I would describe this project ERZgesund as an important breeding ground. For me, for us as a company, it had a certain fundamental work. A breeding ground from which something can develop. In terms of its fundamental work, I would give the whole thing an A+. But what I personally made out of it, I would give it a C- for now, but that’s up to me. […] There is still more to do” (U03, 107).

## 5 Limitations and future research

Strengths and limitations need to be considered in this study. A qualitative method was used to explore an interorganisational WHM network from a company’s perspective, a topic that has not yet been widely studied. One-on-one interviews were conducted to protect anonymity within the network and to encourage open and honest communication. Nevertheless, the qualitative method may have limited the validity of our study’s findings in terms of authenticity or socially desirable response behaviour (57).

Our study provides (in terms of an exploration) initial indicators regarding factors that promote or hinder a WHM network from the companies’ perspective. Moreover, our paper includes an initial form of quantification by reporting the frequencies in addition to the categorisation of content (Table 2). In further studies, the identified indicators should be validated with additional data. On this basis, the generated and validated indicators can be structured and prioritised more specifically (e.g., using the analytical hierarchy process method or regression analysis).

In addition, it must be noted that the recruitment of companies was random, albeit voluntary, meaning companies that had a positive attitude towards the topic were more likely to participate. Furthermore, companies with a low level of participation in the network (one-time participation) were more difficult to reach. Nevertheless, a contrastive sample (SR vs. SP; sector, size, participation in the network) was attempted to map different opinions and perspectives (theoretical saturation). Because the interviews were delayed due to the COVID-19 pandemic, reflections of the ERZgesund network’s advantages or disadvantages might have been overlooked or distorted.

This study focuses on just one case study, the network Erzgesund. Therefore, the generalisability of our results is limited to other WHM networks with different structures and conditions. However, the study shows the first important insights regarding the functionality of a WHM network from the companies’ perspective. Future research should consider other interorganisational collaborations and networks in the field of WHM to expand and validate our findings or to explore other characteristics and (facilitating) factors relating to the functionality of a network from a companies’ perspective. These insights and results could be very helpful for the implementation and further development of WHM networks.

In addition, further analyses should also examine the characteristics of WHM networks in terms of their structures and relationships with the help of social network analyses. This could provide more valuable insights about the companies’ relationships, their mechanisms and characteristics and could provide further information about their perceived perspective.

## 6 Conclusion

The workplace is a promising setting for increasing the population’s health and strengthening companies’ profitability and stability. However, the implementation of WHP is limited, especially in SMEs. Networks such as ERZgesund offer a good opportunity to raise awareness of WHM and to support entry into the implementation of health-promoting interventions. This study provides important initial in-depth results about the functionality of a WHM network from a companies’ perspective. The ERZgesund network succeeded in sensitising companies to WHM, in transferring knowledge, promoting the exchange of experiences and the establishment of new contacts. The regional focus and physical proximity as well as the open setting and level of familiarity among the members were particularly conducive. In addition, the added value was reinforced by the structure of the network with a coordinating body and direct contact person at the top. Therefore, networks are a valuable investment for raising awareness of health and bringing health promotion into companies. Nonetheless, the decision to join a network or implement WHM remains with the company.

However, networks can not only effect change in WHM, but also establish certain relationships, which in turn can offer further benefits such as new orders or support in various areas like sustainability. The network should consciously integrate the companies’ needs and concerns in order to successfully network and realise its goal.

Further studies are required to gain a deeper understanding of the characteristics of WHM networks and the underlying mechanisms by integrating methods of social network analysis and the analytical hierarchy process method. Thus, not only the perceived advantages and disadvantages, but especially the relationships and their effects in the network could be analysed. As a result, the WHM networks could be enhanced and used on a permanent basis, which would benefit not only the participants but also the health awareness of the general public.

## Data availability statement

The raw data supporting the conclusions of this article will be made available by the authors, without undue reservation.

## Ethics statement

The studies were conducted in accordance with the local legislation and institutional requirements (including voluntariness, consent of interviewees, data protection, anonymisation of interview quotes). The participants provided their written informed consent to participate in this study.

## Author contributions

LH: Conceptualization, Data curation, Formal analysis, Funding acquisition, Investigation, Methodology, Project administration, Resources, Visualization, Writing – original draft. TS: Conceptualization, Funding acquisition, Resources, Supervision, Validation, Writing – review & editing, Methodology.
